# Supplementary material for: Integrated Metabolome and Lipidome Strategy to Reveal the Action Pattern of Paclobutrazol, a Plant Growth Retardant, in Varying the Chemical Constituents of Platycodon Root
Source: Molecules. 2022 Oct 14;27(20):6902. doi: 10.3390/molecules27206902 (PMC9609321; doi:10.3390/molecules27206902)
Supplement: Supplementary file 1 [file molecules-27-06902-s001.zip › molecules-1927598-supplementary.pdf]

Supplementary material

Article

Integrated Metabolome and Lipidome Strategy to Reveal the Action Pattern of Paclobutrazol, a Plant Growth Retardant, in Varying the Chemical Constituents of Platycodon Root

Lan Lan [1,†], Weizhen Huang [2,†], Heng Zhou [1], Jiajia Yuan [1], Shui Miao [1], Xiuhong Mao [1], Qing Hu [1] and Shen Ji [1,*]

[1] NMPA Key Laboratory for Quality Control of Traditional Chinese Medicine, Shanghai Institute for Food and Drug Control, Shanghai 201203, China

[2] School of Pharmacy, Yantai University, Yantai 264005, China

* Correspondence: jishen@sifdc.org.cn; Tel.: +86-18001678046

† These authors contributed equally to this work

Table S1 Identification of differential lipids in Platycodon root under the intervention of paclobutrazol

NO.	Compound ID	Adducts	Formula	Mass error/ppm	MS/MS information	Identify	Samples
1	0.93_433.2355*m/z*	[M−H]$^-$	C21H39O7P	−1.34	152.9957, 171.0065, 279.2327	LPA 18:2	3Y
2	0.97_564.3305*m/z*	[M+HCOO]$^-$	C26H50NO7P	−0.32	224.0693, 242.0789,279.2328, 504.3095	LPC 18:2/0:0	3Y
3	7.22_673.4802*m/z*	[M−H]$^-$	C37H71O8P	−1.73	152.9959, 255.2332, 281.2485, 391.2257, 409.2364	PA 16:0/18:1	2Y
4	7.77_699.4965*m/z*	[M−H]$^-$	C39H73O8P	−0.48	283.2643, 415.2260, 419.2567, 437.2677	PA 18:0/18:2	3Y
5	6.39_699.4965*m/z*	[M−H]$^-$	C39H73O8P	−0.23	279.2313,415.2247, 433.2360, 437.2668	PA 18:018:2	3Y
6	4.39_655.4342*m/z*	[M−H]$^-$	C36H65O8P	−0.35	239.2019, 279.2333, 375.1942, 393.2058, 415.2259, 433.2369	PA 18:2/15:1	3Y
7	5.80_697.4810*m/z*	[M−H]$^-$	C39H71O8P	−0.61	279.2331,281.2479, 415.2258, 417.2406, 433.2365, 435.2517	PA 18:2/18:1	2Y
8	4.04_693.4501*m/z*	[M−H]$^-$	C39H67O8P	0.07	152.9959, 277.2176, 279.2330, 413.2099, 415.2253, 431.2208, 433.2361	PA 18:2/18:3	2Y & 3Y
9	5.40_788.5447*m/z*	[M+HCOO]$^-$	C41H78NO8P	0.21	241. 2177, 448.2883, 466.2942, 728.5238	PC 15:0/18:2	3Y
10	4.68_786.5289*m/z*	[M+HCOO]$^-$	C41H76NO8P	−0.20	239.2008, 464.2754, 504.3072, 726.5076	PC 15:1/18:2	2Y
11	6.16_802.5610*m/z*	[M+HCOO]$^-$	C42H80NO8P	0.75	255.2332, 279.2332, 462.2992, 480.3095, 486.2994, 504.3097, 742.5391	PC 16:0/18:2	2Y & 3Y
12	5.09_800.5450*m/z*	[M+HCOO]$^-$	C42H78NO8P	0.38	277.2176, 480.3033, 740.5232	PC 16:0/18:3	3Y
13	7.09_816.5761*m/z*	[M+HCOO]$^-$	C43H82NO8P	0.07	269.2482, 279.2325, 476.3162, 486.3012, 494.3232, 756.5553	PC 17:0/18:2	2Y & 3Y
14	5.92_814.5599*m/z*	[M+HCOO]$^-$	C43H80NO8P	−0.64	267.2316, 486.2990, 492.3091,754.5397	PC 17:1/18:2	3Y
15	6.31_828.5758*m/z*	[M+HCOO]$^-$	C44H82NO8P	0.26	281.2467, 504.3098, 506.3254, 768.5554	PC 18:1/18:2	3Y
16	5.01_698.4763*m/z*	[M−H]$^-$	C38H70NO8P	−0.52	239.2026, 279.2337, 418.2379, 436.2490	PE 15:1/18:2	2Y & 3Y
17	8.27_716.5235*m/z*	[M−H]$^-$	C39H76NO8P	−0.10	255.2336, 281.2490, 434.2681, 452.2795, 460.2847, 478.2954	PE 16:0/18:1	3Y
18	5.08_686.4764*m/z*	[M−H]$^-$	C37H70NO8P	−0.37	227.2007, 424.2482, 476.2792	PE 16:0/18:2	2Y
19	5.48_712.4922*m/z*	[M−H]$^-$	C39H72NO8P	−0.12	196.0377, 277.2168, 434.2407, 452.2786, 456.2571	PE 16:0/18:3	3Y
20	7.64_728.5234*m/z*	[M−H]$^-$	C40H76NO8P	−0.26	269.2480, 279.2323, 448.2839	PE 17:0/18:2	2Y
21	6.36_726.5079*m/z*	[M−H]$^-$	C40H74NO8P	−0.04	267.2326, 279.2325, 464.2792, 476.2816	PE 17:1/18:2	2Y & 3Y
22	8.79_742.5392*m/z*	[M−H]$^-$	C41H78NO8P	0.18	196.0379, 279.2326, 283.2639, 458.2681, 462.2988, 476.2795, 480.3091	PE 18:0/18:2	3Y
23	8.83_742.5394*m/z*	[M−H]$^-$	C41H78NO8P	0.18	196.0379, 279.2326, 283.2639, 458.2681, 462.2988, 476.2795, 480.3091	PE 18:0/18:2	2Y
24	6.79_740.5234*m/z*	[M−H]$^-$	C41H76NO8P	0.08	281.2463, 460.2835, 478.2942	PE 18:1/18:2	3Y
25	5.12_766.5392*m/z*	[M−H]$^-$	C43H78NO8P	0.02	279.2327, 307.2656, 504.3095	PE 18:2/18:2	2Y & 3Y
26	11.63_770.5704*m/z*	[M−H]$^-$	C43H82NO8P	−0.16	279.2326, 311.2951, 476.2791, 490.3301, 508.3406	PE 20:0/18:2	2Y & 3Y
27	13.15_798.6020*m/z*	[M−H]$^-$	C45H86NO8P	0.15	279.2330, 339.3268, 458.2659, 518.3635, 536.3725	PE 22:0/18:2	2Y & 3Y
28	13.66_826.6331*m/z*	[M−H]$^-$	C47H90NO8P	0.32	279.2330, 367.3584, 458.2682, 476.3120, 546.3941, 564.4034	PE 24:0/18:2	3Y

29	13.68_826.6334*m/z*	[M−H]⁻	C47H90NO8P	0.32	279.2330, 367.3584, 458.2682, 476.3120, 546.3941, 564.4034	PE 24:0/18:2	2Y
30	13.12_824.6180*m/z*	[M−H]⁻	C47H88NO8P	0.58	279.23243, 365.34226, 458.26837, 476.27836, 544.37836, 562.38759	PE 24:1/18:2	2Y
31	13.38_838.6333*m/z*	[M−H]⁻	C48H90NO8P	0.26	279.2331, 379.3583	PE 25:1/18:2	2Y & 3Y
32	13.62_852.6487*m/z*	[M−H]⁻	C49H92NO8P	−0.11	279.2325, 393.3738, 590.4208	PE 26:1/18:2	2Y & 3Y
33	5.99_721.5027*m/z*	[M−H]⁻	C38H75O10P	0.23	255.2322, 465.2626, 483.2723	PG 16:0/16:0	2Y
34	6.03_747.5181*m/z*	[M−H]⁻	C40H77O10P	−0.10	281.2473, 491.2779,509.2888	PG 18:1/16:0	2Y & 3Y
35	5.69_835.5340*m/z*	[M−H]⁻	C43H81O13P	−0.23	553.2781, 571.2870, 579.2973	PI 16:0/18:1	3Y
36	3.93_831.5029*m/z*	[M−H]⁻	C43H76O13P	−0.06	223.0004, 241.0119, 277.2169, 391.2258, 553.2785, 571.2896	PI 16:0/18:3	2Y
37	4.76_859.5339*m/z*	[M−H]⁻	C45H81O13P	−0.30	281.2479, 579.2921	PI 18:1/18:2	3Y
38	5.70_711.4968*m/z*	[M−H]⁻	C40H73O8P	0.19	281.2473, 431,2551 449.2674	PMeOH 18:1/18:2	2Y
39	3.96_707.4657*m/z*	[M−H]⁻	C40H69O8P	−0.10	277.2171, 279.2326, 427.3587, 445.2353, 429.2384, 447.2513	PMeOH 18:3/18:2	3Y
40	9.67_741.5437*m/z*	[M−H]⁻	C42H79O8P	−0.35	279.2325, 311.2945, 479.3174	PMeOH 20:/18:2	2Y
41	12.69_769.5751*m/z*	[M−H]⁻	C44H83O8P	−0.18	339.3274, 489.3349, 507.3459	PMeOH 22:0/18:2	2Y
42	13.29_797.6064*m/z*	[M−H]⁻	C46H87O8P	−0.21	279.2315, 367.3560, 535.3772	PMeOH 24:0/18:2	3Y
43	13.00_809.6065*m/z*	[M−H]⁻	C47H87O8P	−0.11	152.9951, 279.2318, 379.3572, 547.3776	PMeOH 25:1/18:2	3Y
44	8.28_814.5603*m/z*	[M−H]⁻	C44H82NO10P	0.55	311.295, 415.2259, 433.2375, 447.2884, 465. 2990, 727.5292	PS 20:0/18:2	3Y
45	8.33_814.5608*m/z*	[M−H]⁻	C44H82NO10P	0.55	311.295, 415.2259, 433.2375, 447.2884, 465. 2990, 727.5292	PS 20:0/18:2	2Y
46	9.52_828.5761*m/z*	[M−H]⁻	C45H84NO10P	0.15	325.3105, 461.3037, 479.3145, 741.5439	PS 21:0/18:0	3Y
47	7.25_826.5603*m/z*	[M−H]⁻	C45H82NO10P	−0.12	267.2330, 323.2955, 415.2253, 433.2737, 459.2880, 477.2990, 739.5287	PS 21:1/18:2	2Y & 3Y
48	10.91_842.5917*m/z*	[M−H]⁻	C46H86NO10P	0.03	339.3266, 415.2255, 433.2374, 475.3204, 493.3307, 755.5599	PS 22:0/18:2	2Y & 3Y
49	12.50_856.6074*m/z*	[M−H]⁻	C47H88NO10P	0.10	353.3426, 489.3355, 507.3463, 769.5785	PS 23:0/18:2	2Y & 3Y
50	13.01_870.6235*m/z*	[M−H]⁻	C48H90NO10P	0.57	367.3575, 415.2255, 433.2362 503.3506, 521.3614, 783.5909	PS 24:0/18:2	2Y & 3Y
51	10.78_868.6072*m/z*	[M−H]⁻	C48H88NO10P	−0.07	279.2323, 365.3413, 415.2239, 501.3345, 519.3451, 781.5750	PS 24:1/18:2	2Y & 3Y
52	12.29_882.6229*m/z*	[M−H]⁻	C49H90NO10P	−0.02	279.2320, 379.3580, 415.2258,515.3508, 533.3615, 795.5914	PS 25:1/18:2	2Y & 3Y

53	16.48_928.8455*n*	[M+NH4]+	C60H112O6	−0.41	575.5023, 649.6127, 673.6117	TG 23:0/18:2/16:0	3Y
54	15.85_936.8130*n*	[M+Na]+	C61H108O6	−1.66	657.5801, 599.5022	TG 22:1/18:2/18:2	2Y
55	16.37_932.8636*m*/*z*	[M+NH4]+	C59H110O6	−0.54	575.5023, 635.5925, 659.5951	TG 22:0/16:0/18:2	3Y
56	15.88_910.7976*n*	[M+Na]+	C59H106O6	−1.43	599.5022, 631.2648	TG 20:0/18:2/18:2	3Y
57	13.41_926.7414*m*/*z*	[M+Na]+	C55H101NO8	−0.60	589.4836, 611.4673	TG 19:0/21:3/16:0	2Y & 3Y
58	13.14_873.6941*m*/*z*	[M+Na]+	C55H94O6	−0.17	595.4709, 597.4863	TG 18:3/18:3/18:3	2Y & 3Y
59	14.49_897.6931*m*/*z*	[M+Na]+	C57H94O6	−1.31	595.4709, 597.4862, 875.7108	TG 18:3/18:2/18:3	3Y
60	15.98_950.8288*n*	[M+Na]+	C62H110O6	−1.49	643.5652, 671. 5959	TG 18:2/21:1/20:2	2Y & 3Y
61	15.71_923.8052*m*/*z*	[M+H]+	C60H106O6	−1.07	643.5651, 599.5030	TG 18:2/21:1/18:2	2Y
62	16.23_978.8604*n*	[M+Na]+	C64H114O6	−1.12	599.5029, 699.6277	TG 18:2/18:2/25:1	3Y
63	16.11_964.8446*n*	[M+Na]+	C63H112O6	−1.35	599.5027, 685.2121	TG 18:2/18:2/24:1	2Y & 3Y
64	14.73_899.7088*m*/*z*	[M+Na]+	C57H96O6	−1.29	597.4868, 599.5013, 877.7268	TG 18:2/18:2/18:3	3Y
65	15.01_901.7243*m*/*z*	[M+Na]+	C57H98O6	−1.43	263.2364, 319.2632, 337.2734, 599.5023	TG 18:2/18:2/18:2	3Y
66	15.59_883.7737*m*/*z*	[M+H]+	C57H102O6	−1.34	599.5029, 603.5330	TG 18:2/18:2/18:0	3Y
67	15.62_905.7557*m*/*z*	[M+Na]+	C57H102O6	−1.28	599.5027, 603.5330,	TG 18:2/18:2/18:0	3Y
68	15.44_868.7506*n*	[M+Na]+	C56H100O6	−1.58	589.5186, 599.5028	TG 18:2/18:2/17:0	3Y

69	14.86_865.7253*m/z*	[M+NH4]+	C56H96O6	−1.92	559.4712, 585.4868	TG 18:2/15:1/20:3	2Y
70	16.62_1012.9259*m/z*	[M+NH4]+	C65H118O6	−0.78	629.5505, 687.6278, 713.6432	TG 18:1/24:1/20:2	3Y
71	15.84_881.7557*m/z*	[M+Na]+	C55H102O6	−1.34	575.5030, 579.5339, 603.5339	TG 18:0/18:2/16:0	3Y: H
72	15.75_896.7826*n*	[M+NH4]+	C58H104O6	−0.75	573.4885, 617.5495, 643.5653	TG 16:1/18:2/21:1	3Y
73	14.90_838.7043*n*	[M+Na]+	C54H94O6	−0.92	585.4868, 559.4712	TG 16:1/17:2/18:2	3Y
74	15.13_840.7208*n*	[M+Na]+	C54H96O6	0.12	587.5017, 561.4862	TG 16:1/17:1/18:2	3Y
75	16.60_942.8611*n*	[M+NH4]+	C61H114O6	−0.45	575.5031, 663.6279, 687.6278	TG 16:0/18:2/24:0	3Y
76	15.95_898.7969*n*	[M+Na]+	C58H106O6	−2.32	575.5029, 619.5653, 643.5652	TG 16:0/18:2/21:1	3Y
77	15.55_853.7243*m/z*	[M+Na]+	C53H98O6	−1.57	551.5022, 575.5024	TG 16:0/18:2/16:0	3Y
78	15.69_844.7511*n*	[M+NH4]+	C54H100O6	−1.04	565.5181, 575.5027, 589.5655	TG 16:0/17:0/18:2	3Y
79	15.80_832.7510*n*	[M+NH4]+	C53H100O6	−1.20	551.5021, 577.5175	TG 16:0/16:0/18:1	3Y
80	15.78_948.8137*n*	[M+NH4]+	C62H108O6	−0.98	597.4873, 669.5809, 671.5959	TG 23:1/18:2/18:3	2Y
81	3.64_613.4819*m/z*	[M+H]+	C39H64O5	−1.30	335.2587, 261.2228	DG 18:3/18:3	2Y
82	4.34_613.4823*m/z*	[M+H]+	C39H64O5	−0.64	261. 2215, 317. 2488, 335.2584	DG 18:3/18:3	2Y & 3Y
83	4.04_615.4975*m/z*	[M+H]+	C39H66O5	−1.34	597.4872, 337.2735, 335.2582, 263.2369, 261.2211	DG 18:3/18:2	2Y
84	4.33_615.4973*m/z*	[M+H]+	C39H66O5	−1.57	261.2215, 335.2584, 337.2739, 597.4872	DG 18:3/18:2	2Y
85	5.21_615.4978*m/z*	[M+H]+	C39H66O5	−0.85	261.2219, 313. 2747, 335. 2587, 337.2739, 597.4868	DG 18:3/18:2	2Y & 3Y
86	7.16_614.4904*n*	[M+Na]+	C39H66O5	−1.09	335.2573, 337.2729, 597.4871	DG 18:3/18:2	3Y
87	6.29_617.5134*m/z*	[M+H]+	C39H68O5	−0.92	599.5024, 337.2730, 263.2208	DG 18:2/18:2	2Y
88	10.92_618.5218*n*	[M+NH4]+	C39H70O5	−0.83	337.2732, 339.2885, 601.5178	DG 18:2/18:1	2Y

89	10.96_641.5110*m/z*	$[M+Na]^+$	C39H70O5	−0.91	337.2732, 339.2885, 601.5178	DG 18:2/18:1	2Y & 3Y
90	8.69_616.5058*n*	$[M+Na]^+$	C39H68O5	−1.48	599.5024, 337.2730, 263.2208	DG 18:1/18:3	3Y
91	14.14_613.4822*m/z*	$[M+H]^+$	C39H64O5	−0.82	261.2220, 277.2170, 313.2745	DG 16:0/18:3	2Y & 3Y
92	14.09_615.4973*m/z*	$[M+Na]^+$	C37H68O5	−1.62	263. 2378, 313. 2745, 337.2742, 575.5034	DG 16:0/18:2	2Y & 3Y
93	10.65_592.5061*n*	$[M+Na]^+$	C37H68O5	−0.94	313.2731, 337.2733, 575.5031	DG 16:0/18:2	2Y & 3Y
94	7.98_634.5767*m/z*	$[M+H]^+$	C40H75NO4	−0.32	616.5656, 598.5550, 336.3265, 298.2737, 262.2525	Cer t18:1/n22:2	2Y & 3Y
95	12.85_708.6491*m/z*	$[M+H]^+$	C44H85NO5	−1.42	262.2529, 280.2636, 298.2735, 316.2852, 690.6387	Cer t18:1/h26:1	2Y: H
96	13.30_707.6418*n*	$[M+H]^+$	C44H85NO5	−1.38	262.2528, 280.263, 298.2737, 672.6281, 690.6388	Cer t18:1/h26:1	2Y & 3Y
97	13.48_710.6648*m/z*	$[M+H]^+$	C44H87NO5	−1.20	262.2529, 280.2636, 298.2735, 412.4158, 674.6400, 692.6550	Cer t18:1/h26:0	2Y
98	13.20_696.6494*m/z*	$[M+H]^+$	C43H85NO5	−0.99	678.6391,660.6275, 398.3978, 380.3908, 316.2842, 262.2527	Cer t18:1/h25:0	2Y
99	13.60_696.6494*m/z*	$[M+H]^+$	C43H85NO5	−0.86	678.6391,660.6275, 398.3978, 316.2842, 298.2738, 280.26324, 262.2527	Cer t18:1/h25:0	3Y
100	10.48_680.6179*m/z*	$[M+H]^+$	C42H81NO5	−1.18	262.2528, 280.2633, 298.2738, 662.6073	Cer t18:1/h24:1	3Y
101	13.56_666.6390*m/z*	$[M+H]^+$	C42H83NO4	−0.75	262.2526, 280.2631, 298.2739, 368.3890, 630.6188, 648.6291	Cer t18:1/h23:1	3Y
102	11.81_668.6182*m/z*	$[M+H]^+$	C41H81NO5	−0.77	650.6073, 632.5959, 370.3678, 298.2739, 280.2631, 262.2526	Cer t18:1/h23:0	2Y & 3Y
103	12.09_668.6183*m/z*	$[M+H]^+$	C41H81NO5	−0.70	650.6073, 632.5959, 370.3678, 298.2739, 280.2631, 262.2526	Cer t18:1/h23:0	3Y
104	13.04_668.6187*m/z*	$[M+H]^+$	C41H81NO5	−0.10	650.6077, 632.6074, 370.3678, 298.2737, 280.2632, 262.2526	Cer t18:1/h23:0	2Y & 3Y
105	9.52_651.5798*n*	$[M+H]^+$	C40H77NO5	−0.62	634.5760, 616.5656, 354.3476, 298.2736, 280.2631, 262.2524	Cer t18:1/h22:1	3Y
106	10.25_654.6026*m/z*	$[M+H]^+$	C40H79NO5	−0.75	636.5917, 618.5816, 356.3520, 298.2737, 280.2630, 262.2525	Cer t18:1/h22:0	2Y & 3Y
107	10.56_654.6026*m/z*	$[M+H]^+$	C40H79NO5	−0.70	636.5917, 618.5814, 600.5715, 356.3523, 298.2739, 280.2633, 262.2524	Cer t18:1/h22:0	2Y
108	12.55_653.5954*n*	$[M+H]^+$	C40H79NO5	−0.59	636.5917, 618.5816, 356.3520, 298.2737, 280.2630, 262.2525	Cer t18:1/h22:0	2Y & 3Y
109	6.93_638.5713*m/z*	$[M+H]^+$	C39H72O5	−0.78	262.2530, 280.2634, 298.2742, 620.5607	Cer t18:1/h21:1	3Y
110	7.70_626.5715*m/z*	$[M+H]^+$	C38H75NO5	−0.54	328.3212, 590.5545, 608.5606	Cer t18:1/h20:0	2Y
111	7.88_626.5716*m/z*	$[M+H]^+$	C38H75NO5	−0.25	634.5762, 616.5656, 354.3361, 298.2737, 280.2630, 262.2525	Cer t18:1/h20:0	2Y
112	4.49_570.5089*m/z*	$[M+H]^+$	C34H67NO5	−0.53	262.2528, 280.2635, 298.2741, 534. 4856, 552.4986	Cer t18:1/h16:0	2Y
113	4.87_534.4879*m/z*	$[M+H]^+$	C34H63NO3	−0.35	516.4769, 504.4773, 298.2752, 280.3633, 262.2523	Cer d18:2/n16:1	2Y
114	5.00_534.4879*m/z*	$[M+H]^+$	C34H63NO3	−0.31	516.4769, 504.4773, 298.2752, 280.3633, 262.2523, 254.2473	Cer d18:2/n16:1	2Y & 3Y
115	5.33_554.5141*m/z*	$[M+H]^+$	C34H67NO4	−0.25	536.5028, 518.4925, 300.2881, 282.2787, 272.2584, 264.2678	Cer d18:1/h16:0	2Y

116	5.47_554.5142*m/z*	$[M+H]^+$	C34H67NO4	−0.07	536.5028, 518.4925, 300.2881, 282.2787, 272.2584, 264.2678	Cer d18:1/h16:0	2Y

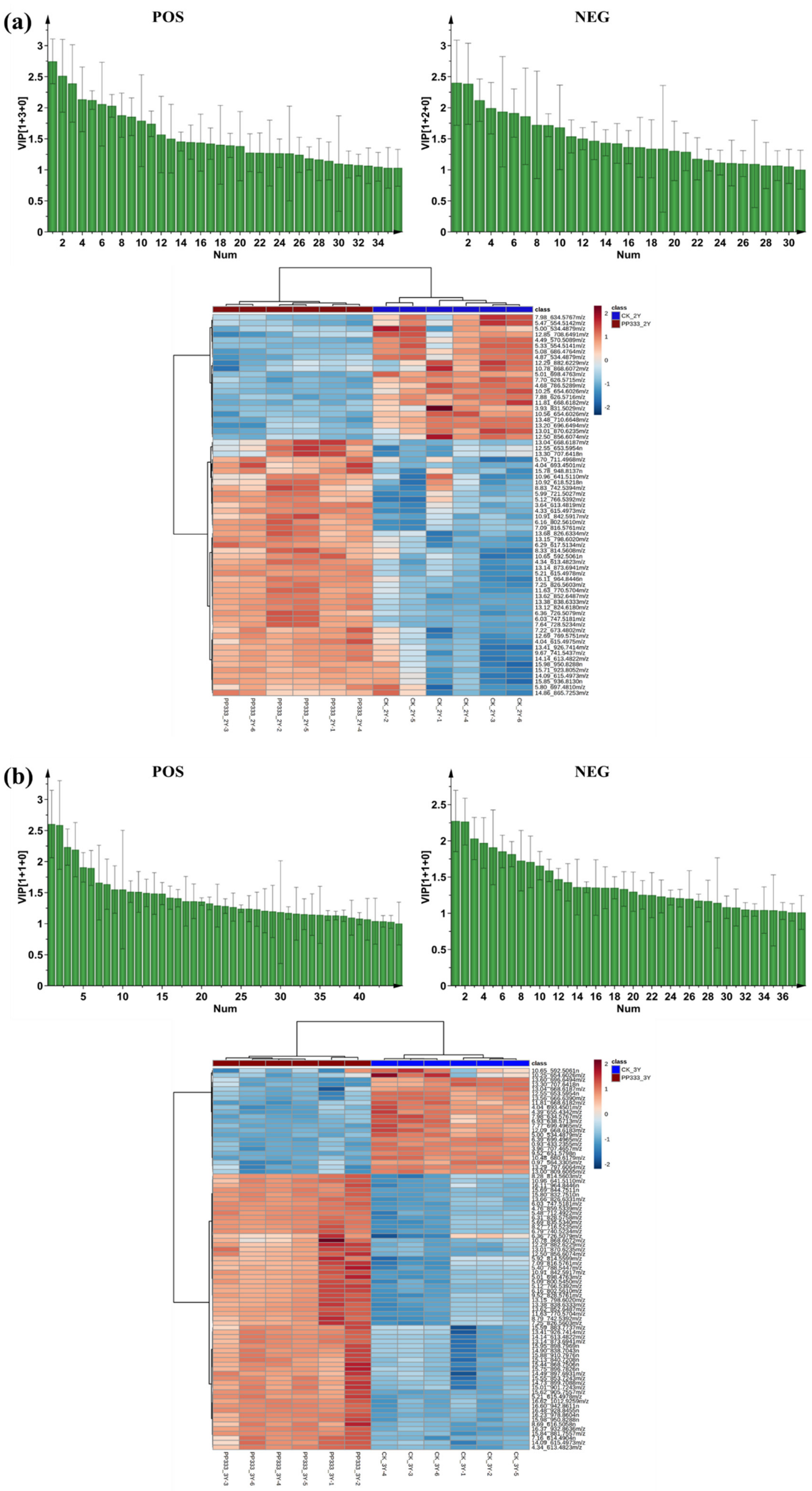

(a)
POS
NEG
VIP[1+3+0]
VIP[1+2+0]
Num
class
CK_2Y
PP333_2Y
PP333_2Y-3
PP333_2Y-6
PP333_2Y-2
PP333_2Y-5
PP333_2Y-1
PP333_2Y-4
CK_2Y-2
CK_2Y-5
CK_2Y-1
CK_2Y-4
CK_2Y-3
CK_2Y-6
(b)
POS
NEG
VIP[1+1+0]
Num
class
CK_3Y
PP333_3Y
PP333_3Y-3
PP333_3Y-6
PP333_3Y-4
PP333_3Y-5
PP333_3Y-1
PP333_3Y-2
CK_3Y-4
CK_3Y-3
CK_3Y-6
CK_3Y-1
CK_3Y-2
CK_3Y-5

Figure S1. VIP plots and heatmaps visualizing the relative content of differential lipids in 2-year-old (a) and 3-year-old (b) samples.

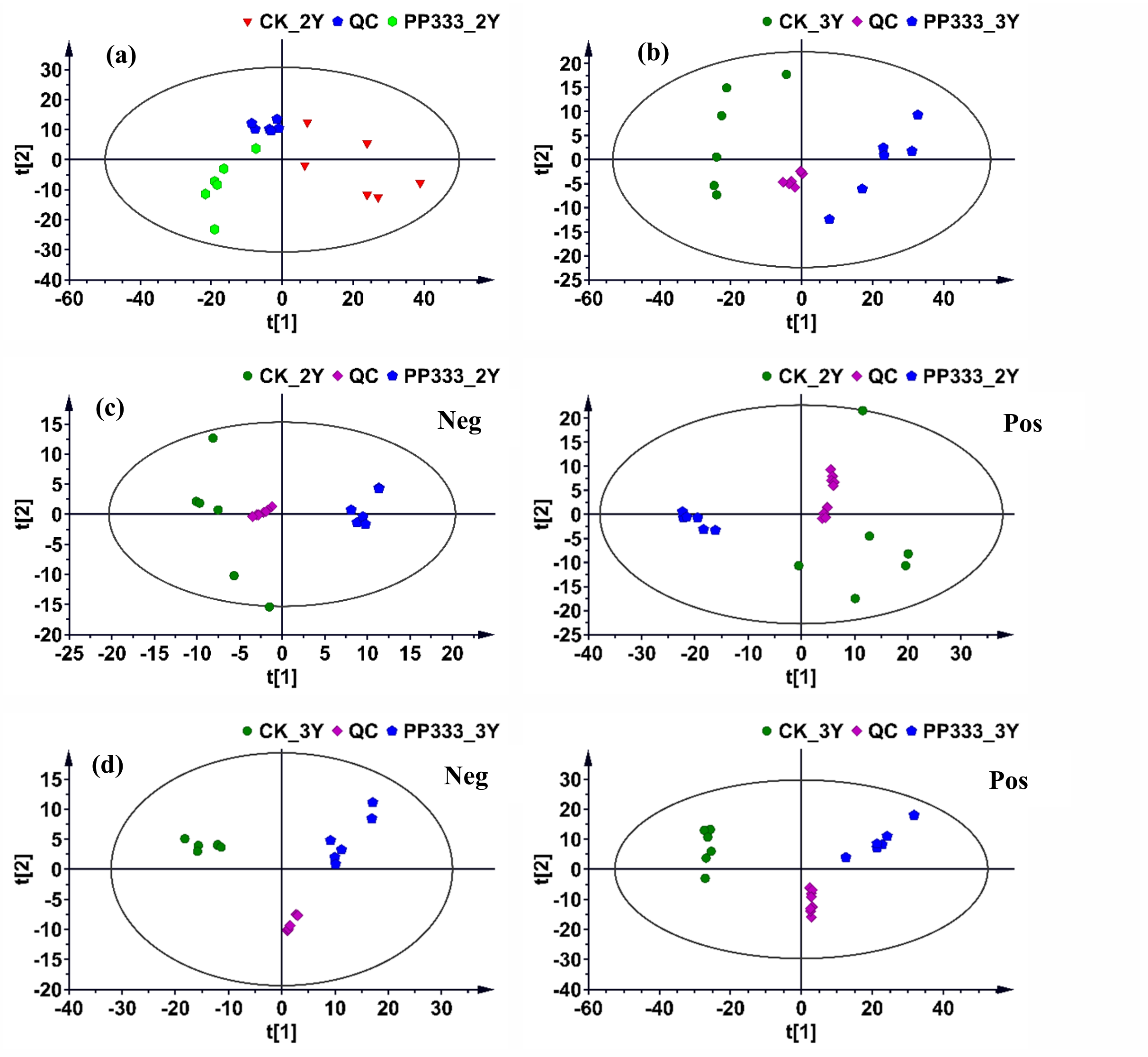

Figure S2. PCA score plot of the metabolome analysis in 2-year-old (a) and 3-year-old (b) Platycodon root. PCA score plots of the lipidome analysis after LC-MS analysis in negative and positive electrospray ionization mode in 2-year-old (c) and 3-year-old (d) Platycodon root. CK_2Y and PP333_2Y represent control and paclobutrazol-treated group of 2-year-old Platycodon root respectively. CK_3Y and PP333_3Y represent control and paclobutrazol-treated group of 3-year-old Platycodon root respectively. QC represents QC sample.
